# Low-Temperature Aging Provides 22% Efficient Bromine-Free and Passivation Layer-Free Planar Perovskite Solar Cells

**DOI:** 10.1007/s40820-020-00418-0

**Published:** 2020-04-03

**Authors:** Xin Wang, Luyao Wang, Tong Shan, Shibing Leng, Hongliang Zhong, Qinye Bao, Zheng-Hong Lu, Lin-Long Deng, Chun-Chao Chen

**Affiliations:** 1 School of Material Science and Engineering, Shanghai Jiao Tong University, Shanghai, 200240 People’s Republic of China; 2 School of Chemistry and Chemical Engineering, Shanghai Jiao Tong University, Shanghai, 200240 People’s Republic of China; 3 Key Laboratory of Solar Materials and Devices, Department of Electronic Science, School of Physics and Electronic Science, East China Normal University, Shanghai, 200240 People’s Republic of China; 4 Department of Materials Science and Engineering, University of Toronto, 184 College Street, Toronto, ON M5S 3E4 Canada; 5 Pen-Tung Sah Institute of Micro-Nano Science and Technology, Xiamen University, Xiamen, 361005 People’s Republic of China

**Keywords:** Aging growth, Bromine-free, Passivation layer, Lead iodide, Perovskite solar cells

## Abstract

Previous reports of formamidinium/methylamine (FAMA)-mixed halide perovskite solar cells have focused mainly on controlling the morphology of the perovskite film and its interface—for example, through the inclusion of bromine and surface passivation. In this paper, we describe a new processing pathway for the growth of a high-quality bromine-free FAMAPbI_3_ halide perovskites via the control of intermediate phase. Through low-temperature aging growth (LTAG) of a freshly deposited perovskite film, α-phase perovskites can be seeded in the intermediate phase and, at the same time, prevent beta-phase perovskite to nucleate. After postannealing, large grain-size perovskites with significantly reduced PbI_2_ presence on the surface can be obtained, thereby eliminating the need of additional surface passivation step. Our pristine LTAG-treated solar cells could provide PCEs of greater than 22% without elaborate use of bromine or an additional passivation layer. More importantly, when using this LTAG process, the growth of the pure alpha-phase FAMAPbI_3_ was highly reproducible.

**Electronic supplementary material:**

The online version of this article (10.1007/s40820-020-00418-0) contains supplementary material, which is available to authorized users.

## Article Highlights

- By low-temperature aging, superior bromine-free FA_1–*x*_MA_*x*_PbI_3_ perovskite film is realized.
- By suppressing lead iodide on the surface of perovskite, no further passivation step or layer is needed. The efficiency of planar perovskite solar cells is improved to 22.41% with robust reproducibility.

**Electronic supplementary material:**

The online version of this article (10.1007/s40820-020-00418-0) contains supplementary material, which is available to authorized users.

## Graphical abstract

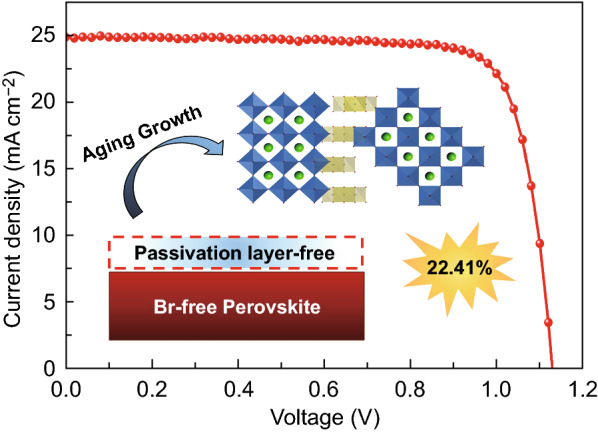

## Introduction

Organic/inorganic hybrid perovskite solar cells (PSCs) are emerging next-generation photovoltaic technologies that have attracted attention for their dramatic improvements in efficiency—from 3.8% [1] to over 24%—in less than a decade [2], their low manufacturing costs, and their ease of solution processing. To promote the device performance of PSCs beyond 20%, several techniques have been investigated, including variations in composition [3–5], control over the perovskite film growth [6–9], interface engineering [10–12], the use of additives [13–15], ionic liquid technique [16], and surface passivation [17–19]. Among the perovskite materials currently being investigated, the low-band-gap α-phase formamidinium lead iodide (FAPbI_3_) perovskite, which possesses an ideal band gap of 1.48 eV and extended light absorption to 840 nm [20–22], appears to have the most promising perovskite composition to achieve record-breaking photocurrent densities and power conversion efficiencies (PCEs) [23–27]. Nevertheless, obtaining the α-phase FAPbI_3_ in its pure composition is difficult through either one-step anti-solvent deposition or the two-step sequential method [15, 28–30]. Therefore, mixed cation and inclusion of bromine have been employed to stabilize the α-phase FAPbI_3_ and, thereby, obtain device performances exceeding 21% [31–33]. Unfortunately, the inclusion of bromine also results in unwanted changes in the composition of the perovskite and contributes to a narrower absorption spectrum. Thus, many attempts have been made toward achieving bromine-free, pure α-phase FAPbI_3_-based solar cells without compromising the intrinsic properties of the FAPbI_3_ perovskite [34–37].

Two-step sequential deposition methods are the most effective methods for preparing FA-based halide perovskite films in planar n–i–p structures [8, 27, 34]. Previous studies have demonstrated that high-quality perovskite films can be obtained in the presence of moderate amounts of humidity [5, 38]. Interestingly, excess lead iodide (PbI_2_) also forms during the growth of perovskite films, which originates from the precursor ingredient and decomposes when high-temperature postannealing of the perovskite film is performed in air [39–41]. Some studies have found that the PbI_2_ by-product has a beneficial effect by passivating the grain boundary defects inside the perovskite film under certain conditions, thereby enhancing device efficiency [32, 40–42]. For example, a small amount of PbI_2_ can passivate the defects on the interface between the perovskite layer (PVK) and the hole-transporting layer (HTL), thus improving device efficiency and eliminating the hysteresis effect [31, 33, 43]. Nevertheless, excess PbI_2_ has negative effects. For instance, if too much PbI_2_ separates out from the perovskite film surface, the thick PbI_2_ layer may itself function as an insulating layer with increased defect density, rather than acting as a passivation layer [44–46]. Therefore, devices prepared with an excess of PbI_2_ often have lower open-circuit voltages (*V*_oc_) and undergo faster photodegradation, compared to PbI_2_-deficient devices [44–48]. Therefore, it is necessary to optimize the amount of PbI_2_ at the PVK–HTL interface to passivate the interface defect states and, thereby, facilitate charge transport.

In several recent studies, efforts have been devoted toward optimizing the passivation layer on the top surface of the perovskite film to remove the excess PbI_2_, by using various passivators, including organic halide salts. For example, Zhu et al. achieved a high value of *V*_oc_ of 1.21 V when using guanidinium bromide salts on a control perovskite film; here, the excess PbI_2_/PbBr_2_ crystals were “digested” with the assistance of the guanidinium bromide [48]. You et al. recovered the contact energy loss and obtained a certified quasi-steady-state efficiency of 23.32% after passivating excess PbI_2_ crystals on perovskite film surface with the organic cationic salt of PEAI [34]. Furthermore, negative effects on the underlying perovskite layer have also been found when an inappropriate solvent or layer thickness was used for the passivation layer [17]. To date, the working mechanism of the passivation layer remains unclear. Determining when to use a passivator, or which passivator to use in a given system, with the hope of reducing the effect of excess PbI_2_ and mitigating non-radiative recombination, can still be a challenge. Developing a passivation layer-free method is important for the fabrication of perovskite layer to be flexible in terms of controlling the ideal amount of PbI_2_ for the self-passivation of surface defects, while also ensuring that the process is simple and reproducible.

In this paper, we proposed a new method, involving low-temperature aging growth (LTAG) prior to thermal annealing, for the production of high-quality bromine-free perovskite films, with control over the composition of PbI_2_, from FA_1–*x*_MA_*x*_PbI_3_ (FA: HC(NH_2_)_2_, MA: CH_3_NH_3_, hereafter denoted “FAMAPbI_3_″) perovskites for planar n–i–p-type solar cells. We demonstrate that an optimized residual content of PbI_2_ on the FAMAPbI_3_ perovskite film surface can be obtained after aging the as-formed perovskite film at low temperature under N_2_ (in a glove box) and annealing in air at a certain relative humidity (RH, 30–40%). Unlike conventional processes that utilize bromine and often lead to a large amount of excess PbI_2_ on the top surface, the perovskite films produced using this LTAG method feature with a fine amount of PbI_2_ crystals between the grain boundaries and much lower PbI_2_ contents on the top surface. Analyses using time-resolved photoluminescence (TRPL) spectroscopy, the space charge-limited current (SCLC) method, ultraviolet photoelectron spectrometry (UPS), and atomic force microscopy (AFM) revealed excellent charge transport and suppressed non-radiative losses at the PVK–HTL interfaces of our LTAG-treated samples. As a result, our champion LTAG devices achieved a record-high PCE-22.41% for a bromine-free FAMAPbI_3_ perovskite. Without the need for additional processing steps to modify the problematic PbI_2_ layer, our present LTAG method can produce suitable amounts of PbI_2_ on the top surface of the perovskite layer to provide stable PSC devices exhibiting high efficiency and excellent reproducibility.

## Experimental Section

### Materials

The SnO_2_ colloid precursor (tin oxide, 15% in H_2_O colloidal dispersion) was purchased from Alfa Aesar. PbI_2_ (99.8%) was purchased from Kanto Chemical. Dimethyl sulfoxide (DMSO 99.8%), chlorobenzene (99.8%), *N*, *N*-dimethylformamide (DMF, 99.8%), and isopropanol (IPA, 99.8%) were obtained from Sigma-Aldrich. Lithium bis(trifluoromethylsulfonyl) imide (Li-TFSI, 95%), 4-*tert*-butylpyridine (TBP, 96%), and 2,2′,7,7′-tetrakis-(*N*, *N*-di-*p*-methoxyphenylamine)-9,9′-spirobifluorene (Spiro-OMeTAD, 99%) were purchased from Lumtec. Formamidinium iodide [CH(NH_2_)_2_I] and methylammonium iodide (CH_3_NH_3_I) were obtained from Dysel. Methylammonium chloride (MACl) was purchased from Xi’an Polymer Light Technology. All chemicals and reagents were used without further purification.

### Solar Cell Fabrication

Patterned ITO glass was cleaned sequentially through sonication in detergent, deionized water, acetone, and isopropyl alcohol. Prior to deposition, the ITO was treated with UV ozone for 15 min. The thin SnO_2_ film was formed by spin-coating onto the substrates from a diluted SnO_2_ precursor solution (2.67%, in water) at 3500 rpm for 30 s; the sample was then annealed in air at 150 °C for 40 min. After cooling to room temperature, the ITO/SnO_2_ substrate was cleaned with UV ozone for 15 min to improve the surface wetting in the following steps. To prepare the perovskite film, 1.5 M PbI_2_ in DMF/DMSO (9:1) was spin-coated on the ITO/SnO_2_ substrate at 1500 rpm for 30 s, and then the sample was annealed at 70 °C for 1 min. A solution of FAI, MAI, and MACl (90:6:9 mg) in IPA (1 mL) was added dropwise onto the PbI_2_ film at 2000 rpm for 30 s; the as-formed fresh film was then converted to a black FAMAPbI_3_ film through annealing on a hot plate in air at 150 °C for 15 min (30–40% humidity). For low-temperature aging growth (LTAG) of the crystallization process, a fresh film was aged in a glove box at various temperatures for 5 min; the as-formed film was transferred into ambient air for further thermal annealing. After the FAMAPbI_3_ perovskite film had formed, the sample was transferred to a glove box and then a solution of Spiro-OMeTAD in chlorobenzene (72.3 mg mL^−1^) containing Li-TFSI solution (17.5 µL; prepared as a 520 mg mL^−1^ solution in MeCN) and TBP (28.8 µL) was spin-coated on the perovskite film at 3000 rpm for 30 s. Finally, a Au film (80 nm) was deposited, as the counter electrode, through thermal evaporation on top of the Spiro-OMeTAD layer. The device size was 0.18 cm^2^; the accurate active cell area was 0.1 cm^2^, using a non-reflective mask, when measuring.

### Characterization

X-ray diffraction (XRD) patterns were obtained using a Rigaku Ultima IV diffractometer and Cu Ka radiation. Scanning electron microscopy (SEM) images were obtained using a JEOL JSM-7800F Prime scanning electron microscope. UV–Vis absorption spectra were recorded using a Lambda 35 UV–Vis spectrometer. Steady-state fluorescence and time-resolved photoluminescence (TRPL) spectra were recorded using a FLS1000 photoluminescence spectrometer with excitation at a wavelength of 450 nm. Current density–voltage (J–V) curves of the photovoltaic devices were measured using a Keithley 2400 source meter. Photocurrents were measured under AM1.5G illumination at 100 mW cm^−2^ using an Abet Technologies Sun 2000 solar simulator. External quantum efficiency (EQE) spectra were recorded using an Enlitech LQE-50-FL IPCE measurement system. The light intensity was calibrated using a silicon standard. The active area of each cell was 0.1 cm^2^. The *J*–*V* measurements were recorded in ambient air without encapsulation; both reverse (1.2 → − 0.1 V) and forward (− 0.1 → 1.2 V) scans were performed using the same step of 0.02 V s^−1^. Electrochemical impedance spectroscopy (EIS) was performed using a Donghua DH7000 electrochemical workstation. Ultraviolet photoelectron spectroscopy (UPS) was performed using an AXIS Ultra DLD instrument with the emitting UV energy at 21.21 eV.

## Results and Discussion

### Fabrication and Characterization of the Perovskite Films

**Fig. 1:**
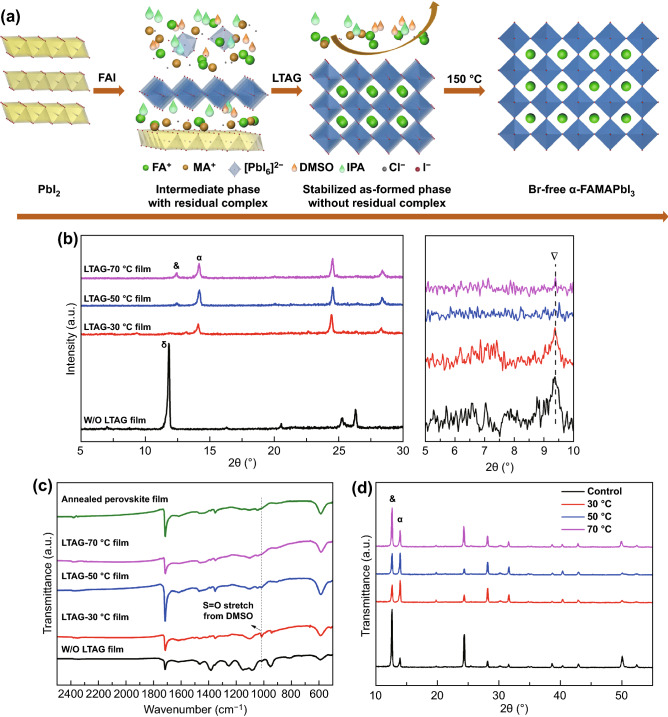
**a** Schematic representation of the film crystallization process of an LTAG-based FAMAPbI_3_ perovskite layer. **b** XRD patterns of the as-formed films obtained using the control process and the LTAG method. &, *α*, *δ*, ∇ Mean characteristic diffraction signals of the PbI_2_, black perovskite, yellow perovskite phases, and intermediate phase, respectively. **c** FTIR spectrum of the as-formed film powers using the control process and the LTAG method and for postannealed FAMAPbI_3_ film. **d** XRD patterns of annealed perovskite layers prepared without (control) and with LTAG treatment (at 30, 50, and 70 °C)

In the low-band-gap FAMAPbI_3_ perovskite systems, a robust preparation method without bromine is highly desirable to provide devices with higher photocurrent. A new two-step sequential method is presented here to deposit a bromine-free FAMAPbI_3_ perovskite absorber film. As displayed in Fig. 1a, in the conventional two-step method, PbI_2_ is dissolved in a mixed solvent of DMF and DMSO. The first step involves formation of a PbI_2_–DMSO film. In the second step, a solution of the mixed organic FAI and MAI is applied, such that critical intramolecular exchange occurs with the PbI_2_–DMSO film to form an intermediate-phase FAI–MAI-PbI_2_–DMSO film [26]. Typically, the brown fresh film is directly subjected to high-temperature annealing in air at a controlled humidity (RH, 30–40%) to allow the highly crystallized perovskite films to form [6]. However, the freshly prepared intermediate film usually transforms, within approximately 10 s, into a yellow *δ*-phase FAMAPbI_3_ when exposed to air, resulting in a white or opaque surface for final perovskite film (Fig. S1). As reported previously [49], the intermediate phase is a layered-structure film that lacks an exact composition. If it is not handled properly, this intermediate phase is highly unstable which can cause problems in the following perovskite crystallization process.

In our LTAG method, as displayed in Fig. 1a, the freshly coated intermediate-phase brown film is first left on a hot plate and aged at various low temperatures for 5 min. Specifically, the low-temperature aging is set at 30, 50, and 70 °C for LTAG-30, LTAG-50, and LTAG-70, respectively. Then, the as-formed aged film is then baked on a hot plate at 150 °C for 15 min to produce the *α*-phase perovskite film (Fig. S2). To study the mechanism of LTAG process, we performed XRD patterns of the as-formed intermediate-phase films for control and LTAG method (at 30, 50, and 70 °C) after the exposure to air but prior to final high-temperature annealing, as shown in Fig. 1b. For the control film without LTAG treatment, the intermediate phase peaked at 9.3° is clearly observed [29, 50]. The undesired *δ*-phase perovskite peaked at 11.6° is, however, dramatically high, indicating the existence of δ-phase perovskite inside intermediate film. In LTAG-based film, as displayed in Fig. 1b, no signal of *δ*-phase FAMAPbI_3_ is found at 11.6°. The signals at 9.3° and 13° for LTAG-30 indicate that the intermediate phase is well kept for the aging temperature at 30 °C. More importantly, *α*-phase FAMAPbI_3_ peaked at 13.94° starts to appear in the intermediate phase after LTAG treatment, indicating LTAG can precrystallize *α*-phase perovskite [51–53]. With increases of aging temperature to 50 and 70 °C, the PbI_2_ peak at 12.7° starts to exist. This shows that the intermediate phase is volatile and can easily turn into yellow *δ*-perovskite phase in the control film, but with the help of LTAG the intermediate film is more likely to nucleate α-phase perovskite.

To further certify this phenomenon, Fourier transform infrared spectroscopy (FTIR) was performed and results are shown in Fig. 1c. The characteristic peak in 1016 cm^−1^ represents S = O stretch, indicating the intermediate FAI–MAI-PbI_2_–DMSO phase from the coordination between DMSO and MA^+^, FA^+^, and Pb^2+^ ions [29, 50]. For control film, the intermediate FAI-MAI-PbI_2_-DMSO phase exists, and the intensity of intermediate phase decreases when the aging temperature increases and disappears at the temperature of 70 °C. These results indicate that LTAG process can avoid the formation of yellow *δ*-phase FAMAPbI_3_ as compared to control process. The conversion of the intermediate-phase film to *α*-phase perovskite is more thorough by releasing the residual complex compositions (e.g., IPA, MAI, or MACl) in the intermediate film beforehand. In other words, LTAG process can help the intermediate phase to start nucleating *α*-phase perovskite at low aging temperature and finish the conversion to *α*-phase perovskite during high-temperature postannealing.

Figure 1d presents XRD patterns of the postannealed perovskite films prepared using various aging temperatures. All of the samples exhibited characteristic peaks at 12.7° and 13.94°, which were assigned to PbI_2_ and α-phase perovskite, corresponding to their (001) and (100) reflections, respectively (Fig. S3) [7]. The intensity of the PbI_2_ diffraction peak was remarkably high in the XRD pattern of the control sample. In this case, the PbI_2_ diffraction peak was even stronger than that of the perovskite peak. For the LTAG samples, the intensity ratios of the PbI_2_ (001) and perovskite (100) signals were much lower than that for the control sample. For low-temperature aging at 30 °C, the intensity of the PbI_2_ peak was the lowest among all of our tested samples. The intensity of the PbI_2_ peak got stronger with increasing temperature of LTAG method, which was also in consistence with the previous results from XRD and FTIR in the as-prepared films. In other words, the existence of *δ*-phase in the as-prepared intermediate phase from control process may cause an incomplete conversion of *δ*-phase to *α*-phase perovskites, thereby inducing mass nucleation of PbI_2_ in the meantime. When using the LTAG method, the *α*-phase perovskites already precrystallized in the as-prepared intermediate films can help form highly crystallized *α*-phase perovskite in postannealing step and, at the same time, reduce the presence of PbI_2_, which is critically important for the later preparation of high-performance devices [54].

**Fig. 2:**
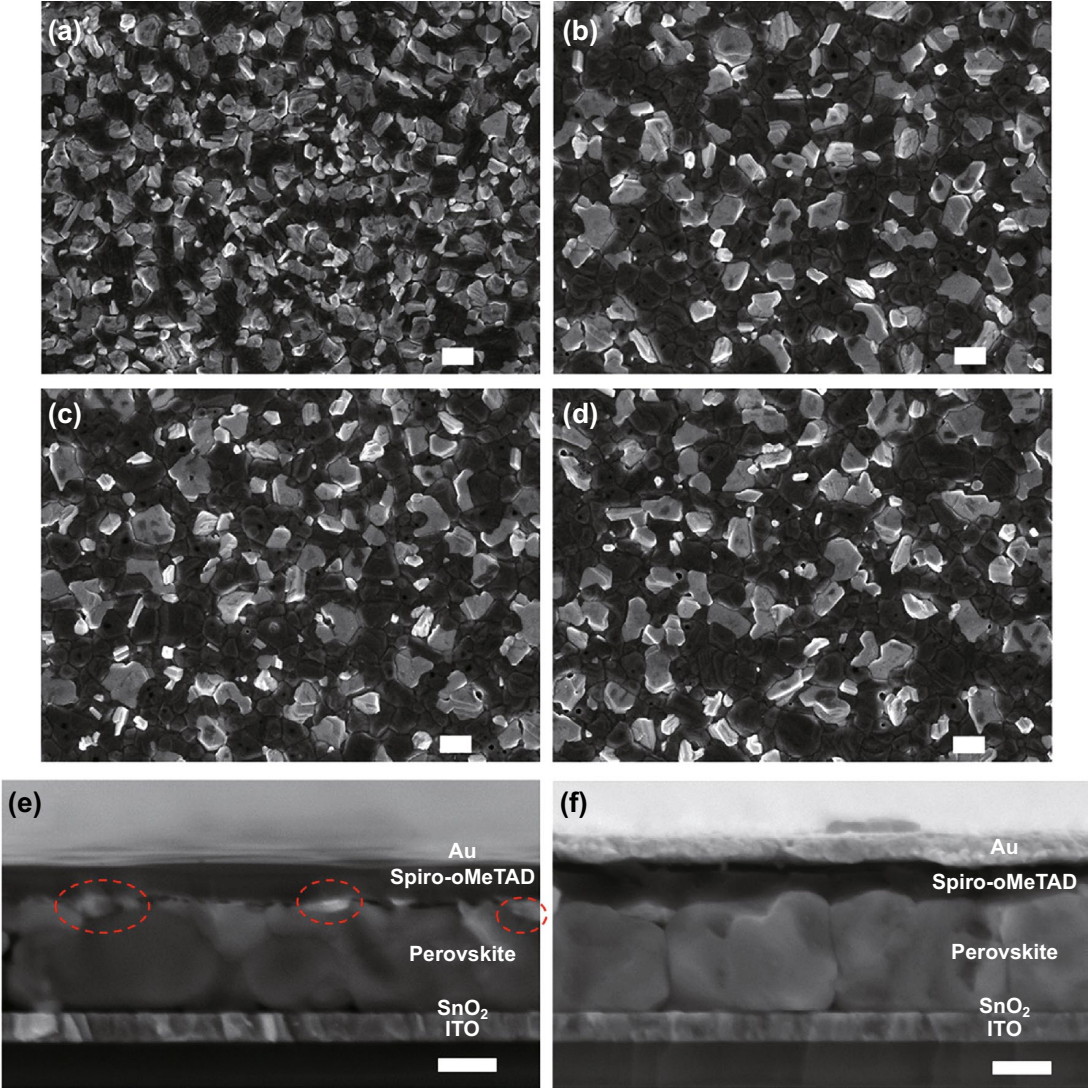
Top-view SEM images of postdeposition annealed perovskite films: **a** control sample without aging, **b** LTAG-30-treated sample, **c** LTAG-50-treated sample, and **d** LTAG-70-treated sample. Cross-sectional SEM images of **e** control and **f** LTAG-30-treated device; the scale bars: 1 μm (**a–d**); 500 nm (**e, f**)

To examine the amount of excess PbI_2_, we recorded SEM images of the top surfaces of thermally postannealed perovskite films—one prepared without aging, hereafter denoted as the control sample/film, and three prepared using the LTAG method with aging at temperatures of 30, 50, and 70 °C, hereafter denoted as LTAG-30, LTAG-50, and LTAG-70 samples/films, respectively. In Fig. 2a, for the sample prepared without intermediate film aging, large amounts of bright crystals were present on the surface. In these bright crystals, the dense white areas were formed by PbI_2_, while the dark areas were the surrounding perovskite grains [6, 31]. For the LTAG-based perovskite films (Fig. 2b–d), the numbers of white PbI_2_ crystals decreased dramatically. The remaining PbI_2_ was distributed around the perovskite grain, suggesting a suppressed PbI_2_ formation when using the LTAG method. The LTAG-30 sample featured the minimum amount of PbI_2_ on the top surface; here, the grain size of the perovskite had increased to 1.15 μm (Fig. S4). The average grain-size distribution obtained from SEM images is exhibited in Fig. S5, indicating that LTAG treatment can restrain the PbI_2_ contents and induce a larger grain size of perovskite crystals as compared to that of control samples. These results are also in consistence with the cross-sectional SEM images for control and LTAG-30-treated devices in Fig. 2e, f, an excess amount of PbI_2_ is evident on the top surface in control sample, but no PbI_2_ is visible on the top surface for LTAG-30 device. In other words, during the thermal postannealing recrystallization process, the formation of the perovskite crystals and the PbI_2_ crystals occurred simultaneously. The LTAG method appeared to seed *α*-phase perovskites in the intermediate phase and allow the α-phase perovskite crystal grains to grow into larger sizes, thereby limiting the formation of PbI_2_ at the surface. In addition, the red shift of absorption edges of LTAG-based perovskite films was observed (Fig. S6). The visible band gap of the LTAG-based films was slightly shifted to a narrower band gap (ca. 1.53 eV). Similar shift has also been found when the content of PbI_2_ was decreased on perovskite layer [33].

### Device Fabrication and Characterization

**Fig. 3:**
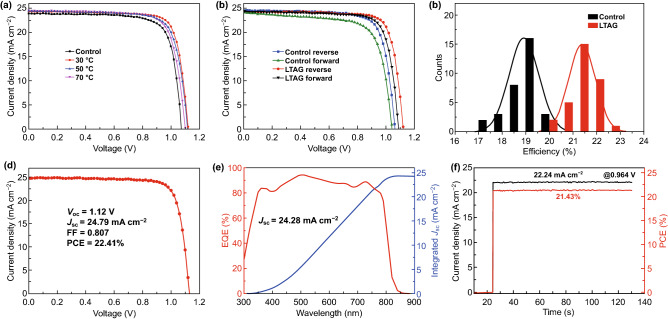
**a** *J*–*V* curves of devices prepared using the LTAG method (at 30, 50, and 70 °C), compared with that of the control device. **b** *J*–*V* curves recorded from reverse and forward scans for the control device and the LTAG-30 solar cell. **c** Distributions of PCEs measured from the control and LTAG-30 devices. **d** *J*–*V* curve of the best-performing LTAG-30 perovskite solar cell. **e** EQE spectrum and integrated value of *J*_sc_ of the best-performing LTAG-30 device. **f** Stabilized current density and power output of the best-performing LTAG-30 device, measured close to the maximum power point

We investigated the efficiencies of solar cells incorporating the perovskite films prepared under the various conditions. The control device and the LTAG-based devices, prepared using aging temperatures of 30, 50, and 70 °C, were fabricated with the following planar configuration: indium tin oxide (ITO)/SnO_2_/FAMAPbI_3_/Spiro-OMeTAD/Au. The thicknesses of the SnO_2_, FAMAPbI_3_ perovskite, and Spiro-OMeTAD layers, determined from cross-sectional SEM images, were 35, 800, and 150 nm, respectively (Fig. S7). Figure 3a presents the current density–voltage (J–V) curves plotted with respect to the aging temperature.

Table 1 summarizes the average photovoltaic parameters obtained from 32 devices subjected to the aging treatment. When the aging temperature was set at 30 °C for LTAG-30, the average PCE of perovskite devices reaches the best performance of 21.4%. Further increase in aging temperature to 50 and 70 °C did not continue to increase the performance but did not keep efficiency at 20%, which is still greatly enhanced from that of the control devices at 18.9% efficiency. Furthermore, 30 °C is the optimal LTAG temperature attributing to its capability to keep the intermediate phase without drying it completely. The proof of this suggestion can be found in Fig. 1b. The control devices provided a low PCE of 19.75%, an open-circuit voltage (*V*_oc_) of 1.06 V, a short-current current density (*J*_sc_) of 23.96 mA cm^−2^, and a fill factor (FF) of 0.77. The LTAG-based devices displayed significantly improved photovoltaic performance, particularly in terms of their values of *V*_oc_ and *J*_*sc*_. For example, the value of *J*_sc_ of the LTAG-30 sample could exceed 24.40 mA cm^−2^—the highest measured in this study. The values of *V*_oc_ obtained from the devices incorporating the LTAG-treated films that had been aged at 30, 50, and 70 °C were 1.10, 1.12, and 1.10 V, respectively.

**Table 1:** Average and champion device performance parameters for solar cells processed using the controlled process (control) and the LTAG process (30, 50, 70)

Aging temperature (°C)	*V*_oc_ (V)	*J*_sc_ (mA cm^−2^)	FF	PCE (%)	Champion PCE (%)
Control	1.06 ± 0.02	23.5 ± 0.4	0.75 ± 0.03	18.9 ± 0.6	19.75
30	1.12 ± 0.01	24.4 ± 0.4	0.79 ± 0.01	21.4 ± 0.5	22.41
50	1.10 ± 0.02	24.2 ± 0.2	0.76 ± 0.02	20.2 ± 0.7	20.91
70	1.10 ± 0.02	24.1 ± 0.4	0.75 ± 0.02	19.9 ± 0.7	20.67

Thirty-two devices were fabricated for each condition

Furthermore, we studied the time effect of LTAG (Figs. S8, S9, and Table S1). It was found that 5-min aging time is required to complete LTAG and obtain reduced PbI_2_ crystals. When aging time is prolonged over 5 min, there exhibited no significant difference on the morphology and corresponding device performance. These results suggest that decreasing and precisely controlling the PbI_2_ content significantly affected the device performance. Considering that a fine amount of PbI_2_ could act as a passivation agent for the grain boundary defects but not affect charge transport at the PVK–HTL contact, we could use the LTAG-30 device, which exhibited the highest performance, to define the optimal content of residual PbI_2_ in this study. Indeed, the LTAG-30 device exhibited the highest PCE of 22.41%, with a *V*_oc_ of 1.12 V, a *J*_sc_ of 24.79 mA cm^−2^, and an FF of 0.807 (Fig. 3d). Compared with the results reported previously for FAPbI_3_-based solar cells prepared without passivation layers (Table S2), this device efficiency is the highest ever recorded for a bromine- and passivation layer-free planar-structure perovskite solar cells. Furthermore, we tested the device performance when LTAG-based film was annealed in the ambient under different humidity conditions and found that the corresponding device performance exhibited almost no decrease, indicating a better stability toward humidity as compared to the control devices which are found with a significant degradation under high humidity environment (Fig. S10).

We also tested the devices obtained after performing the aging growth under various atmospheres (N_2_, Ar, and O_2_); Figure S11 and Table S3 present the corresponding photovoltaic parameters, which reveal that the aging growth treatment was the key factor, rather than the N_2_ atmosphere, promoting the greater device performance [55]. We attribute the enhancement in device performance to the lower residual PbI_2_, the enhanced light absorption, and the improved charge recombination, as inferred from the XRD, absorption spectral, and PL intensity data.

To examine the hysteresis effects, we measured the J–V curves of the LTAG-based and control devices in reverse and forward scans. As shown in Fig. 3b, the LTAG-30 device exhibited lower hysteresis, along with a lower H-index, relative to the control device (Table S4), suggesting superior charge transfer from the PVK to the HTL in the LTAG-30 sample. Figure 3c presents the PCE distributions of the devices obtained using the LTAG and control methods. Table S5 summarizes the detailed parameters of the LTAG-30 cells. We obtained PSCs with a PCE of approximately 22% and high reproducibility, for each of the devices obtained using the LTAG method. Figure 3e displays the external quantum efficiency (EQE) spectra and the integrated values of *J*_sc_ of the LTAG-30 champion device. The calculated value of *J*_sc_ of 24.24 mA cm^−2^ matches well with the value measured (24.79 mA cm^−2^) from the J–V curves, higher than that of the control device (Fig. S12). Figure 3f reveals a stabilized steady-state efficiency of 21.43%, with a steady-state current density of 22.24 mA cm^−2^ at a constant bias voltage of 0.96 V, for the best-performing LTAG-30 device. Figure S13 provides the corresponding data for the reference device. Furthermore, we examined the device stability at room temperature of the un-capsulated perovskite solar cells stored in a dry oven (RH = 20%; Fig. S14). The LTAG-30 device retained 90% of its initial PCE after 1800 h; in contrast, the control device retained only 70% of its initial PCE after the same duration. Accordingly, we conclude that decreasing the amount of residual PbI_2_ at the interface improved the device stability, possibly also by mitigating the ion migration of PbI_2_ across the interface.

**Fig. 4:**
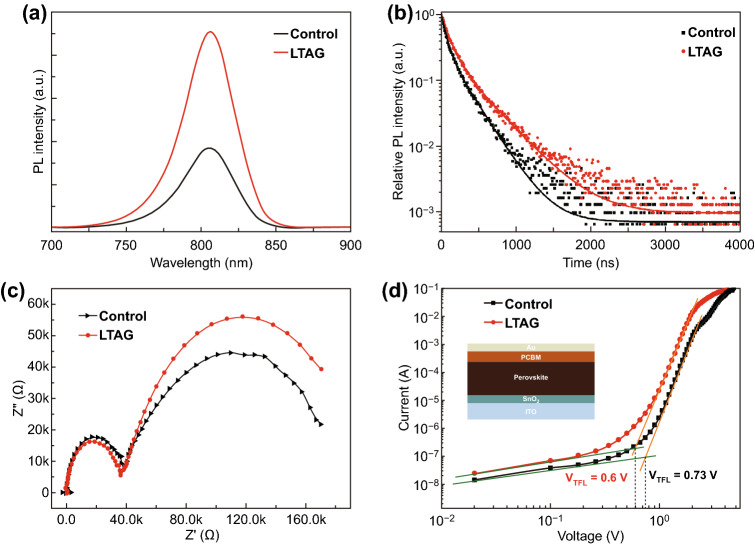
**a** Steady-state PL spectra of annealed perovskite layers prepared without (control) and with LTAG treatment at 30 °C. **b** TRPL spectra of the control and LTAG-based perovskite films. **c** Nyquist plots for the control and LTAG-based perovskite devices in the dark. **d** Current–voltage curves and extracted defect densities of electron-only devices prepared using the control and LTAG-based methods; insets: the single-carrier device structures

To investigate the dynamics of the photocarriers in these devices, and their relationship with the improvement in the value of *V*_oc_, we recorded steady-state PL spectra of the thermally postannealed perovskite films. Figure 4a reveals that the PL intensity increased dramatically for the LTAG-30-based film relative to the control sample, implying improved photocharge properties and a decrease in the amount of spontaneous non-radiative recombination from the surface traps in the LTAG samples. We also measured the TRPL spectrum (Fig. 4b) as dots, which we fitted using a biexponential decay function (solid lines) featuring fast (*τ*_1_) and slow (*τ*_2_) decay times [56]. We derived average lifetime constants of 497.81 and 846.03 ns for the control and LTAG-30 films, respectively (Table S6). The LTAG-based film possessed the longer average lifetime, indicating a decrease in its non-radiative recombination and potentially passivated surface traps, arising from the well-controlled residual PbI_2_ content across the perovskite layer surface. We used EIS to study the interfacial charge transfer properties. As displayed in Fig. 4c, the high and low frequencies corresponded to the bulk transfer (*R*_tr_) and interface recombination (*R*_rec_) resistances in the perovskite device, respectively [57]. Compared with the control sample, we measured a lower value of *R*_tr_ and a higher value of *R*_rec_ in the LTAG sample (Fig. S15, Table S7), suggesting that charge transfer across the bulk material and the contact interface had both improved in the LTAG-based device.

We used the SCLC method to examine the decrease in the defect density for our devices, employing the following electron-only device configuration: ITO/SnO_2_/FAMAPbI_3_/PC_61_BM/Au. Figure 4d reveals three different regions in the logarithmic I–V curves: a linear ohmic region (slope = 1), a nonlinear trap-filling region (slope > 2), and a SCLC region (slope = 2) [58]. Upon increasing the bias voltage in region 2, the trap-filled limit voltage (*V*_TFL_) was reached when all of the traps were filled, and the defect density (*N*_t_) could be calculated from Eq. 1 [58]:

1 $$N_{\text{defect}} \, = \,2\varepsilon_{0} \varepsilon_{r} V_{\text{TFL}} /eL^{2}$$

where *ε*_r_ is the relative permittivity of perovskite material (*ε*_r_ = 32) [9, 59], *ε*_0_ is the vacuum permittivity (*ε*_0_ = 8.85 × 10^−12^ F m^−1^), *e* is the electron charge, and *L* is the thickness of perovskite film (*L* = 800 nm) in the devices [60]. The value of *V*_TFL_ decreased from 0.73 to 0.60 V after performing the aging treatment; the estimated defect density of 4.97 × 10^15^ cm^−3^ for the LTAG-based device was lower than that for the control reference (6.05 × 10^15^ cm^−3^). This finding suggests that effective self-passivation had occurred and, thus, the number of traps had decreased. This phenomenon contributed to the observed higher values of *V*_oc_, which led to the higher PCEs, of the LTAG-based solar cells [61].

Energy band alignment between the perovskite and transport layers is crucial to avoiding non-radiative recombination and *V*_oc_ loss at the interface [62, 63]. To examine the intrinsic cause of the improvement in the value of *V*_oc_ after the aging growth treatment, we used UPS to examine the electronic structure of the perovskite layers. Figure S16 presents the electronic properties of the control sample featuring an excess of residual PbI_2_ and of the LTAG sample featuring the fine residual PbI_2_. From the cutoff region, we found that the work functions (WFs) increased from − 4.0 V for the control film to − 3.9 eV for the LTAG-based film. The valence band energies (*E*_VB_) of control and LTAG-based films were − 5.91 and − 5.86 eV, respectively. The slight increase in the value of *E*_VB_ upon decreasing the amount of residual PbI_2_ presumably led to a better match with the reported value of Spiro-OMeTAD (− 5.22 eV), resulting in enhanced hole transport from the perovskite to the HTL and minimized carrier recombination [64], both contributing partially to the improved values of *V*_oc_ of our LTAG-based devices.

**Fig. 5:**
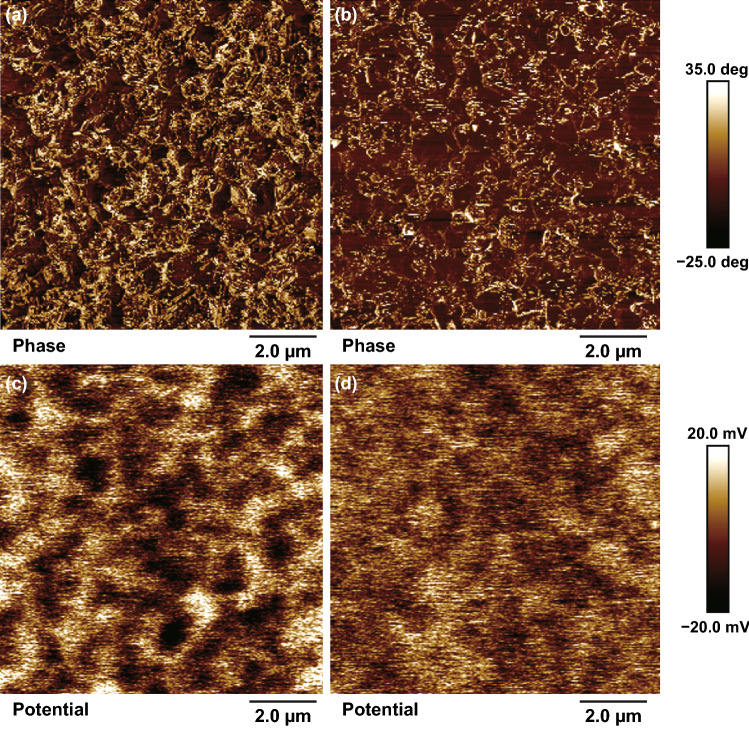
**a, b** AFM phase images and **c, d** 2D surface potential spatial maps of **a, c** the control perovskite film and **b, d** the perovskite film prepared through LTAG at 30 °C

To identify the surface defects, we applied conductive atomic force microscopy (c-AFM) in dark to detect the phase and potential differences of the perovskite films. Figure 5a, b presents AFM phase images of the control and LTAG films, respectively. For the control film, the white PbI_2_ phases had aggregated and were common on the top surface. In contrast, in the LTAG film, the crystal grains of the perovskite (observed with a dark color) were common on the top surface, with the white PbI_2_ phase likely present in the grain boundary areas. Figure 5c, d presents two-dimensional (2D) surface potential spatial maps of the control and LTAG films, respectively. The potential distribution in the control perovskite film is discontinuous and surrounded by the separated white areas, suggesting high surface potential barriers exist between perovskite crystals and grain boundaries due to the excess amount of PbI_2_ on the perovskite surface. However, the film provided by the LTAG method shows a relatively uniform potential distribution across the perovskite surface without any remarkable dots, indicating that a minimized surface potential barrier exists between the perovskite crystals and grain boundaries. The potential barrier between the perovskite grain and grain boundary can cause a significant influence on the carrier transfer [65]. For the control film, the surface potential of the separated white areas of PbI_2_ was 40 mV higher than that of the dark areas of the bulk perovskite, indicating severe charge trapping in the PbI_2_ areas. For the LTAG film, the film surface potential difference between the PbI_2_ and the bulk perovskite was much lower, at 15 mV, revealing that the self-passivation of PbI_2_ was highly effective and that charge trapping at the grain boundaries had been alleviated. This improvement resulted from the decreased presence of PbI_2_ phases on top of the perovskite phases, enhancing the carrier transporting capability [39, 66]. These results are consistent with our experimental observations from the TRPL, EIS, SCLC, and UPS analyses, which suggested that LTAG treatment improved the values of *V*_oc_ and, thereby, the performance of the photovoltaic devices.

## Conclusions

We have demonstrated that preannealing through an LTAG method produces highly crystallized and bromine-free FAMAPbI_3_ perovskite films, with control over the content of PbI_2_. In LTAG method, the composition of as-prepared intermediate film is optimized by inhibiting the formation of *δ*-phase perovskite and allowing α-phase perovskite to nucleate. Thereby, when postannealing is applied, seeded α-phase perovskites can easily turn into large grain-sized perovskites with suppressed PbI_2_ formation. This process readily optimizes the levels of PbI_2_ at the interface between the perovskite and the HTL and at the grain boundaries, resulting in effective self-passivation of grain boundary defects and lowering of the PVK/HTL contact resistance. Moreover, the corresponding LTAG-based devices exhibited superior PCEs of up to 22.41% with increased *V*_oc_ of up to 1.12 V, high reproducibility and low hysteresis. The small residual amount of PbI_2_ created through the LTAG process was beneficial to decreasing the recombination loss on the top perovskite surface without requiring the use of an additional passivation layer or passivation agents, which may have negative effects. Therefore, this method of preannealing low-temperature aging is a simple process that provides highly efficient and reproducible low-band-gap FAMAPbI_3_ PSCs.

## Electronic Supplementary Material

Below is the link to the electronic supplementary material.

Supplementary material 1 (PDF 1737 kb)
