# Supplementary material for: Low-Temperature Aging Provides 22% Efficient Bromine-Free and Passivation Layer-Free Planar Perovskite Solar Cells
Source: Nanomicro Lett. 2020 Apr 3;12:84. doi: 10.1007/s40820-020-00418-0 (PMC7770720; doi:10.1007/s40820-020-00418-0)
Supplement: Supplementary file 1 — Supplementary material 1 (PDF 1737 kb) [file 40820_2020_418_MOESM1_ESM.pdf]

Supporting Information for

Low-Temperature Aging Provides 22% Efficient Bromine–Free and Passivation Layer-Free Planar Perovskite Solar Cells

Xin Wang[1], Luyao Wang[1], Tong Shan[2], Shibing Leng[1], Hongliang Zhong[2], Qinye Bao[3], Zheng-Hong Lu[4]*, Lin-Long Deng[5]*, Chun-Chao Chen[1]*

[1]School of Material Science and Engineering, Shanghai Jiao Tong University, Shanghai 200240, People's Republic of China

[2]School of Chemistry and Chemical Engineering, Shanghai Jiao Tong University, Shanghai 200240, People's Republic of China

[3]Key Laboratory of Solar Materials and Devices, Department of Electronic Science, School of Physics and Electronic Science, East China Normal University, Shanghai 200240, People's Republic of China

[4]Department of Materials Science and Engineering, University of Toronto, 184 College Street, Toronto, ON, M5S 3E4, Canada

[5]Pen-Tung Sah Institute of Micro-Nano Science and Technology, Xiamen University, Xiamen 361005, People's Republic of China

*Corresponding authors. E-mail: zhenghong.lu@utoronto.ca (Zheng-Hong Lu), denglinlong@xmu.edu.cn (Lin-Long Deng), c3chen@sjtu.edu.cn (Chun-Chao Chen)

Supplementary Figures and Tables

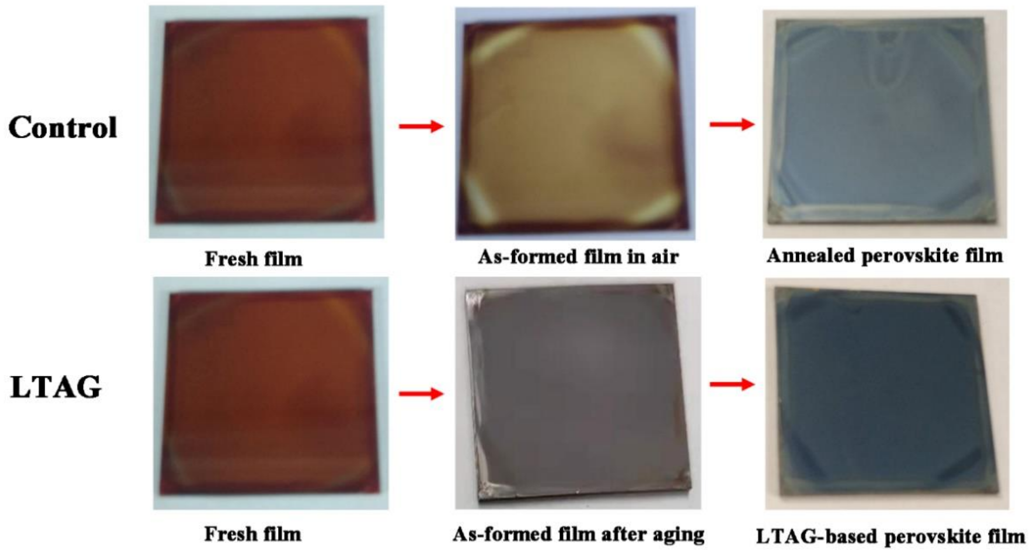

Fig. S1 Photographs of perovskite films at various steps of the fabrication process, prepared using the control and LTAG-based processes

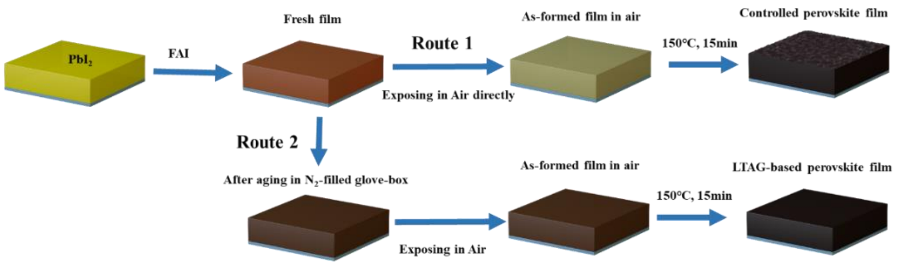

Fig. S2 Schematic representation of the fabrication processes of perovskite films. Route 1: The control process without aging, resulting in too much PbI_2 on the perovskite surface. Route 2: The LTAG method, resulting in a lower amount of PbI_2 on the PVK surface

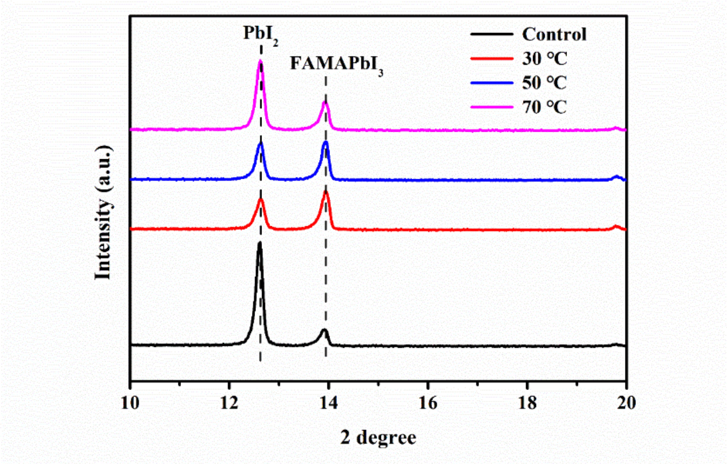

Fig. S3 XRD patterns revealing the PbI_2 (001) and perovskite (100) peaks obtained after applying the control process and the LTAG process at aging temperatures of 30, 50, and 70 °C

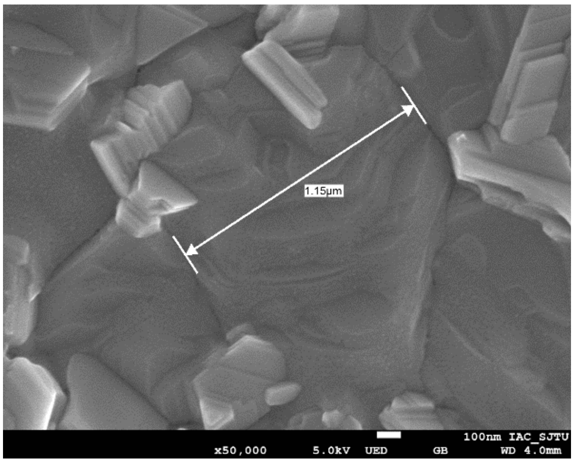

Fig. S4 High-power scanning electron microscopy (SEM) image of the perovskite film obtained after aging treatment at 30 °C, revealing relatively large grains with sizes of up to 1.15 μm

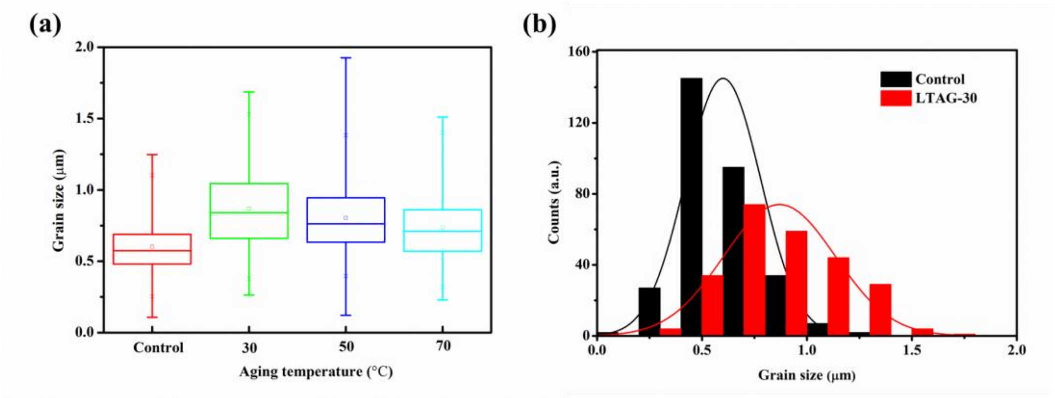

Fig. S5 (**a**) Grain size distribution obtained from SEM images for perovskite films. Average perovskite size for the film without (control) and LTAG-treated films (at 30, 50, and 70 ° C) are 606 nm, 871 nm, 810 nm, 730 nm, respectively. (**b**) Grain statistics and Guassian distribution fitting for control and LTAG-30 based films

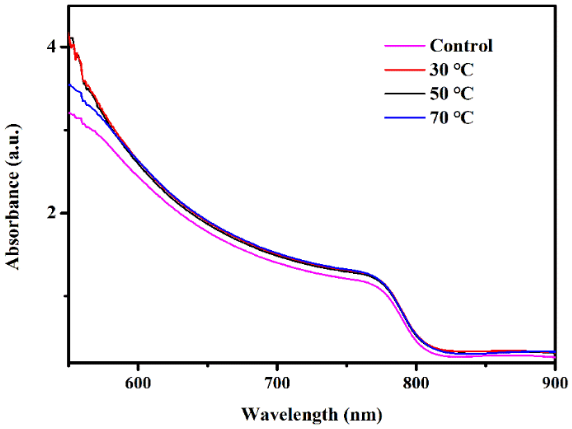

Fig. S6 Absorption spectra of $FAMAPbI_3$ films prepared without (control) and with LTAG treatment (at 30, 50, and 70 °C). Perovskite with LTAG-treated film shows a red shift near 810nm, indicating the visible band gaps of the LTAG-based $FAMAPbI_3$ perovskite films was slightly shifted to narrow bandgap (ca. 1.53 eV) with the decreased PbI_2 contents.

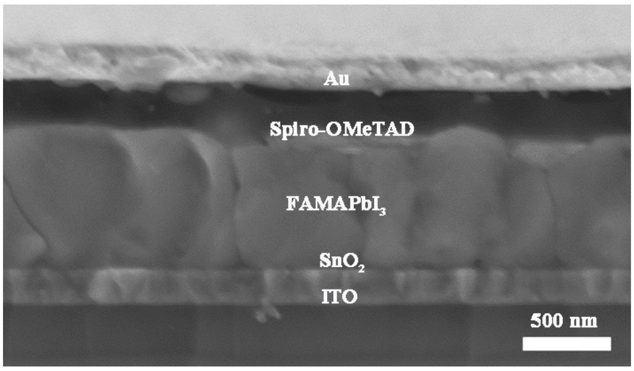

Fig. S7 Cross-sectional SEM image of a completed perovskite device having the structure glass/ITO/SnO_2/$FAMAPbI_3$/Spiro-OMeTAD/Au

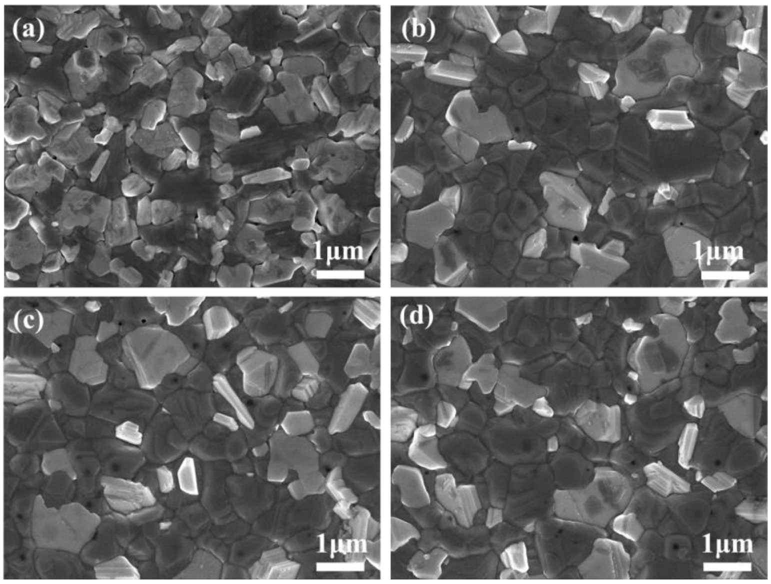

Fig. S8 Top-view SEM images of annealed perovskite films with low-temperature aging process at varies time for (**a**) 2 min, (**b**) 5 min, (**c**) 10 min, and (**d**) 15 min

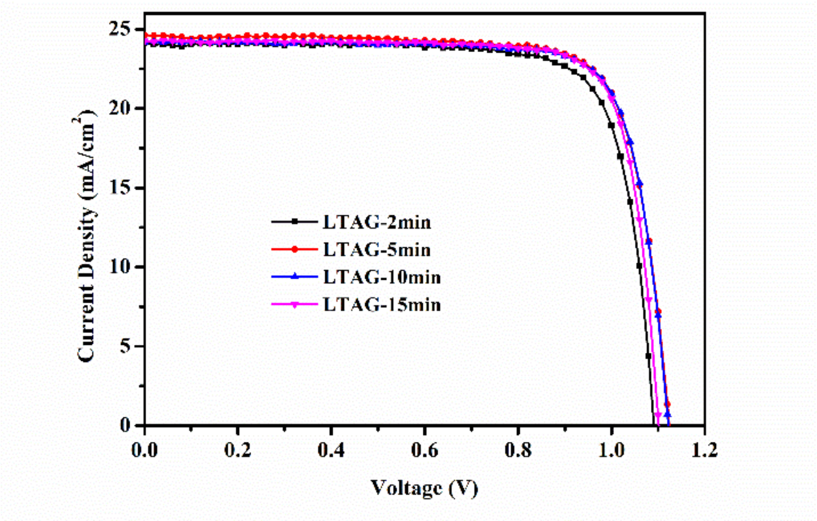

Fig. S9 *J*–*V* curves of devices prepared using LTAG method with different aging time (at 2 min, 5 min, 10 min and 15 min)

Table S1 Device performance parameters for solar cells with low-temperature aging processed at varies aging time

Aging time	V_{oc} (V)	J_{sc} (mA cm^{-2})	FF (%)	PCE (%)
2 min	1.08	24.12	79.16	20.63
5 min	1.12	24.62	78.20	21.57
10 min	1.12	24.24	79.25	21.52
15 min	1.10	24.31	80.03	21.40

Table S2 Reported results for passivation layer–free $FAPbI_3$-based planar PSCs

ETL	Perovskite composition	Br content	PCE (%)	Year	Refs.
SC-CBD SnO_2	$Cs(FAPbI_3)_{0.83}(MAPbBr_3)_{0.17}$	17 %	20.7	2016	[S1]
SnO_2	$(FAPbI_3)_{0.97}(MAPbBr_3)_{0.03}$	3 %	20.51	2016	[S2]
TiO_2-Cl	$FA_{0.85}MA_{0.15}PbI_{2.55}Br_{0.45}$	15 %	21.4	2017	[S3]
SnO_2@a-TiO_2	$FA_{0.85}MA_{0.15}PbI_{2.55}Br_{0.45}$	15 %	21.1	2017	[S4]
SnO_2	$MA_{0.03}FA_{0.97}Pb(I_{0.97}Br_{0.03})_3$	3 %	21.6	2017	[S5]
C_9-SnO_2	$(FAPbI_3)_x(MAPbBr_3)_{1-x}$	10 %	21.3	2018	[S6]
SnO_2+KOH	$Cs_{0.05}(FA_{0.85}MA_{0.15})_{0.95}Pb(I_{0.85}Br_{0.15})_3$	15 %	20.5	2018	[S7]
SnO_2	$Cs_{0.02}MA_{0.03}FA_{0.95}Pb(I_{0.95}Br_{0.05})_3$	5 %	22.0	2019	[S8]
NH_4Cl-SnO_2	$(FAPbI_3)_{0.97}(MAPbBr_3)_{0.03}$	3 %	21.38	2019	[S9]
ZnO	$FA_{0.83}Cs_{0.17}Pb(I_{0.83}Br_{0.17})_3$	17 %	21.1	2019	[S10]
SnO_2	$(FAPbI_3)_{95}(MAPbBr_3)_5$-MACl	5%	22.51	2019	[S11]
SnO_2-KCl	$(FAPbI_3)_x(MAPbBr_3)_{1-x}$	3%	22.2	2020	[S12]
SnO_2	$(FAPbI_3)_{0.92}(MAPbBr_3)_{0.08}$	8%	22.6	2020	[S13]
PTAA	$Cs_{0.05}(FA_{0.92}MA_{0.08})_{0.95}Pb(I_{0.92}Br_{0.08})_3$	8%	22.3	2020	[S14]
SnO_2	$FA_{0.95}Cs_{0.05}PbI_3$	**Br-free**	21.6	2018	[S15]
SnO_2	$FA_{1-x}MA_xPbI_3$		21.24	2019	[S16]
TiO_2	$FA_{0.15}MA_{0.85}PbI_3$		21.38	2019	[S17]
In_2O_3/SnO_2	$FA_{1-x}MA_xPbI_3$		22.54	2020	[S18]
SnO_2	$FA_{1-x}MA_xPbI_3$		**22.41**	2020	This work

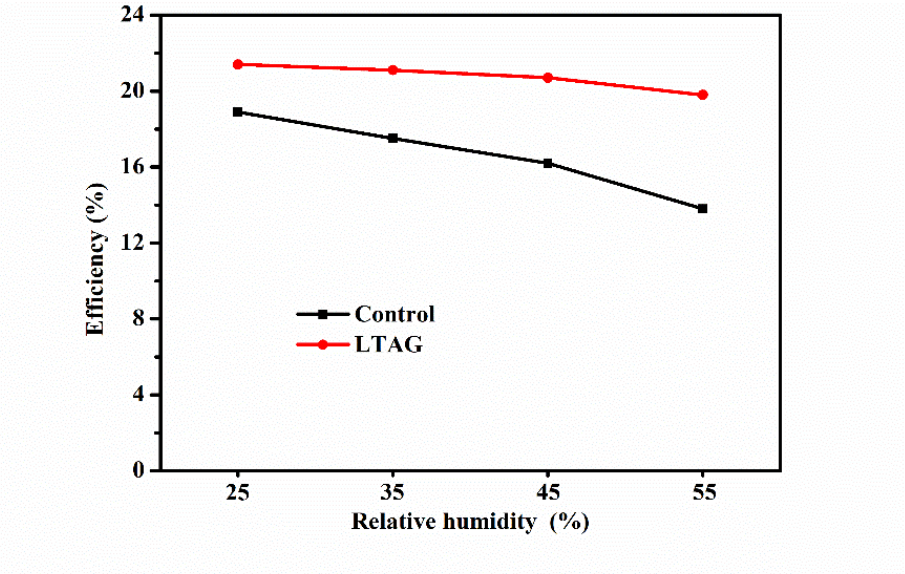

Fig. S10 Effects of relative humidity on control (without LTAG) and LTAG-based perovskite devices

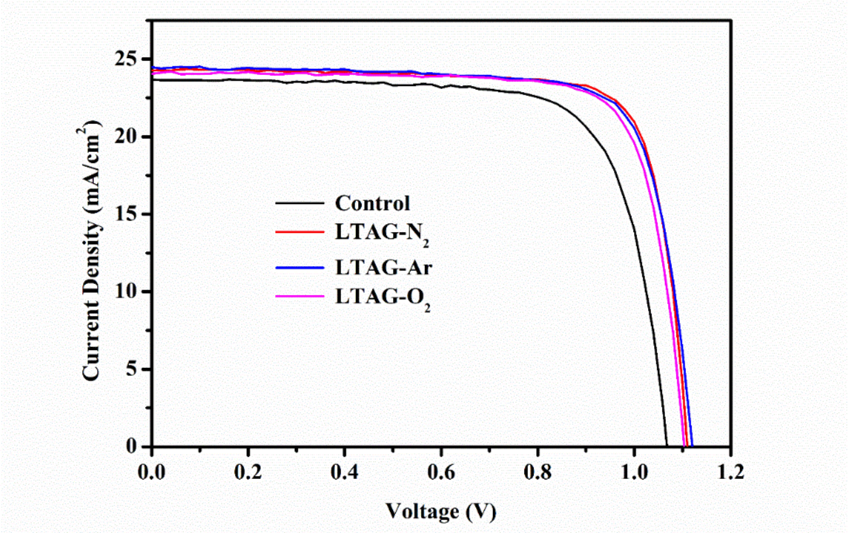

Fig. S11 *J–V* curves for perovskite samples prepared through LTAG treatment under various gases

Table S3 Device performance parameters obtained from the *J–V* curves of PSCs prepared through LTAG treatment under various gases

LTAG-Perovskite	V_{oc} (V)	J_{sc} (mA cm^{-2})	FF (%)	PCE (%)
Control	1.06	23.70	74.47	18.71
N_2	1.12	24.28	78.95	21.47
Ar	1.12	24.52	77.50	21.28
O_2	1.1	24.21	78.42	20.89

Table S4 Photovoltaic parameters obtained from the *J–V* curves (both reverse and forward scans) of the control device and the LTAG-based solar cells. H-index was calculated using the equation H-index = ($PCE_{reverse}$ – $PCE_{forward}$)/$PCE_{reverse}$, where $PCE_{reverse}$ and $PCE_{forward}$ are the PCEs measured in the reverse and forward scan directions

	Scan Direction	V_{oc} (V)	J_{sc} (mA cm^{-2})	FF (%)	PCE (%)	H-index (%)
Control	Reverse	1.06	23.78	76.98	19.41	10.2
	Forward	1.04	23.50	71.32	17.43	
LTAG	Reverse	1.12	24.39	80.34	21.95	3.1
	Forward	1.1	24.29	79.58	21.26	

Table S5 J–V parameters of LTAG-30 devices recorded under illumination of 1 sun (AM1.5, 100 mW cm^{-2})

Sample	V_{oc} (V)	J_{sc} (mA cm^{-2})	FF (%)	PCE (%)
1	1.12	24.38	79.27	21.64
2	1.12	24.63	78.207	21.57
3	1.12	24.24	79.257	21.52
4	1.10	23.86	79.85	20.95
5	1.10	24.71	80.15	21.78
6	1.14	24.42	78.89	21.94
7	1.12	24.39	80.35	21.95
8	1.10	24.29	79.59	21.26
9	1.12	24.79	80.70	22.40
10	1.08	24.80	76.75	20.56
11	1.12	24.40	76.29	20.84
12	1.12	24.52	77.50	21.28
13	1.12	24.28	78.95	21.46
14	1.10	24.41	79.06	21.23
15	1.12	24.14	80.05	21.64
16	1.12	23.61	79.67	21.06
17	1.10	23.08	78.89	20.03
18	1.12	24.77	79.34	22.01
19	1.10	24.31	80.03	21.40
20	1.08	24.12	79.16	20.63
21	1.10	22.71	80.14	20.02
22	1.08	24.51	78.95	20.89
23	1.10	24.67	81.38	22.08
24	1.10	24.60	78.53	21.25
25	1.12	24.35	79.98	21.82
26	1.13	24.44	80.52	22.24

27	1.10	24.56	80.04	21.62
28	1.12	24.82	78.00	21.68
29	1.10	24.65	79.42	21.72
30	1.11	24.60	78.56	21.45
31	1.10	24.53	77.88	21.02
32	1.10	24.29	79.59	21.26

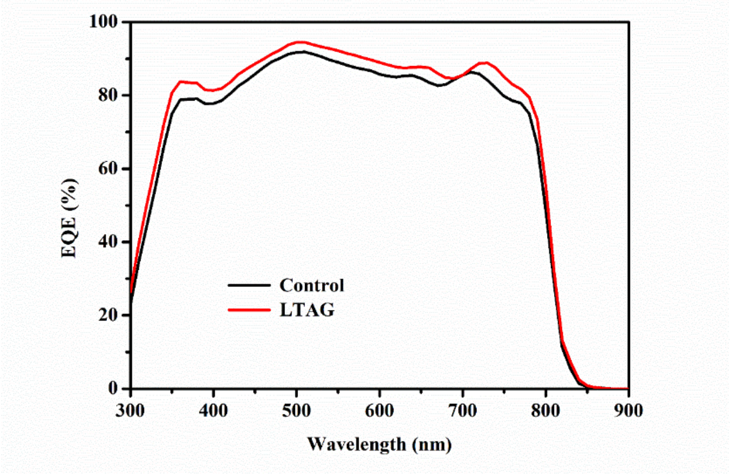

Fig. S12 External quantum efficiency (EQE) spectra of the control device and the 30 °C-aged device featuring $FAMAPI_3$ as the absorber. The current densities for the control and LTAG-based devices, calculated from these curves, are 23.33 and 24.24 mA cm^{-2}, respectively. Both values match well with the current densities measured from the *J*–*V* curves

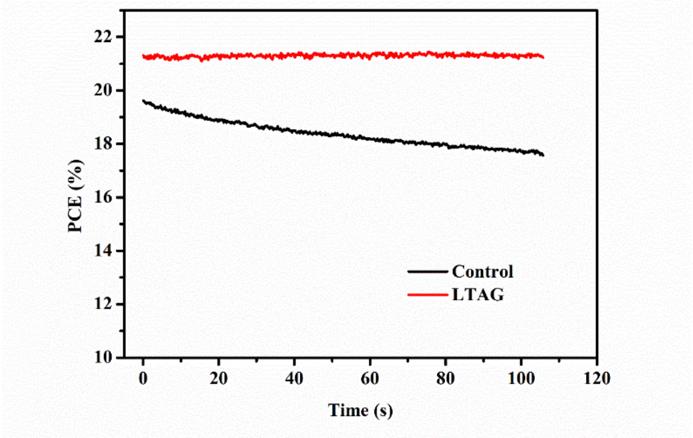

Fig. S13 Steady state power outputs of the champion control and LTAG-based devices, measured close to the maximum power point. The stabilized current densities for the control and LTAG devices were 21.2 and 22.24 mA cm^{-2}, respectively; their maximum voltages were 0.92 and 0.96 V, respectively. The steady state output increased from 19.5 to 21.4% after applying the LTAG process.

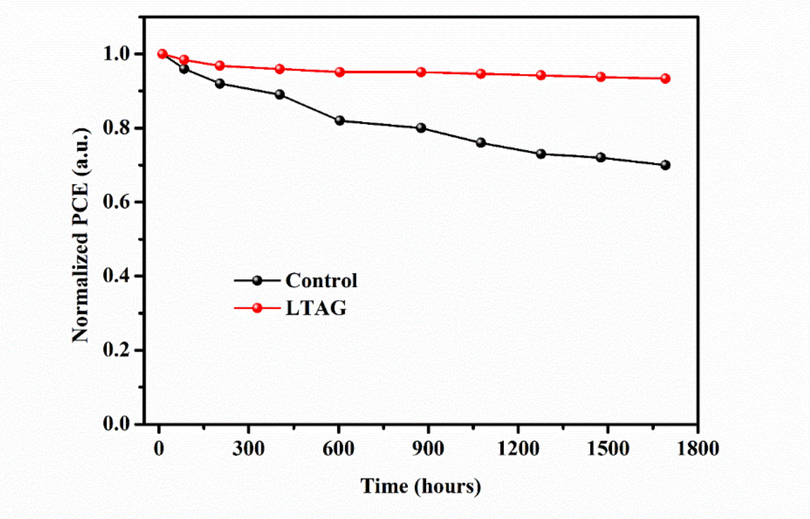

Fig. S14 Storage stability tests for the control and LTAG-based perovskite devices, placed in a dry oven without encapsulation

Table S6 Parameters obtained from time-resolved photoluminescence (TRPL) spectra of the control and LTAG-based perovskite films

	τ_1 (ns)	A_1 (%)	τ_2 (ns)	A_2 (%)	τ_{ave} (ns)
Control	157.59	47.03	579.9	52.97	497.81
LTAG	201.51	70.19	1119.26	29.81	846.03

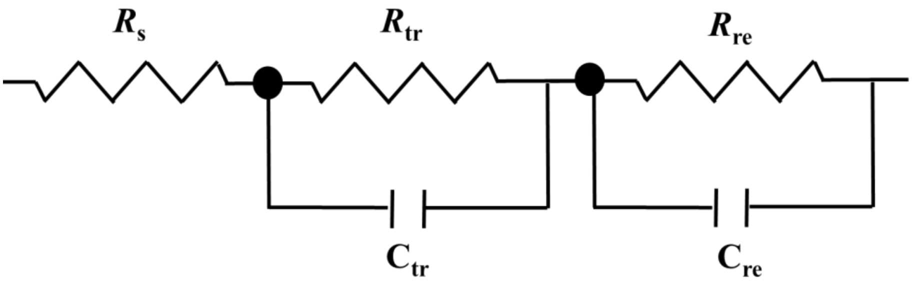

Fig. S15 Equivalent circuit model for EIS. The equivalent circuit composed of series resistance and charge-recombination resistance

Table S7 EIS parameters for the control and LTAG-based perovskite devices

Perovskite	R_{tr} (Ω)	R_{re} (Ω)
Control	32665	146900
LTAG	32325	170500

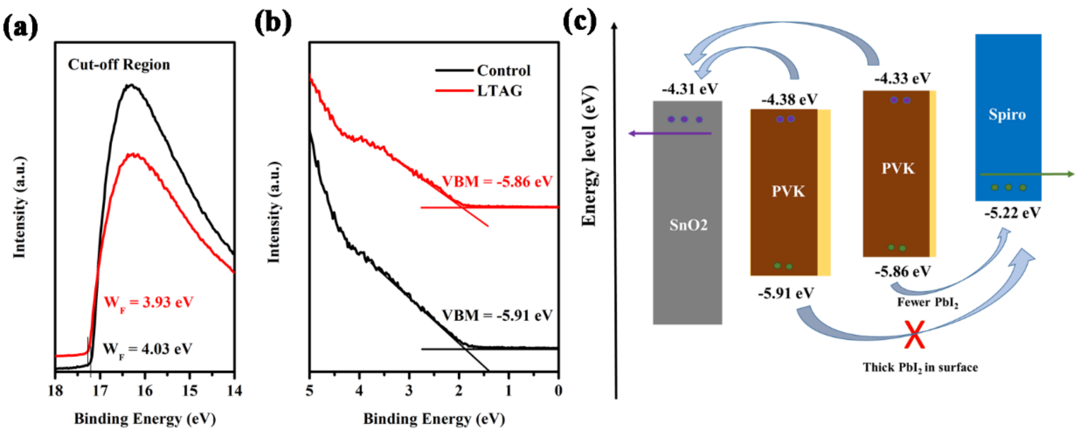

Fig. S16 (**a**) Secondary electron cut-off region of the UPS spectra of the control and LTAG devices. (**b**) Valence band region of the UPS spectra of the control and LTAG devices. (**c**) Energy band alignments for perovskite films prepared with and without aging treatment

Supplementary References

[S1]E.H. Anaraki, A. Kermanpur, L. Steier, K. Domanski, T. Matsui et al., Highly efficient and stable planar perovskite solar cells by solution-processed tin oxide. Energy Environ. Sci. **9**(10), 3128-3134 (2016). https://doi.org/10.1039/C6EE02390H

[S2]Q. Jiang, L. Zhang, H. Wang, X. Yang, J. Meng, Enhanced electron extraction using SnO_2 for high-efficiency planar-structure $HC(NH_2)_2PbI_3$-based perovskite solar cells. Nat. Energy **2**, 16177 (2016). https://doi.org/10.1038/nenergy.2016.177

[S3]H. Tan, A. Jain, O. Voznyy, X. Lan, F.P. García de Arquer, J.Z. Fan et al., Efficient and stable solution-processed planar perovskite solar cells via contact passivation. Science **355**(6326), 722-726 (2017). https://doi.org/10.1126/science.aai9081

[S4]S. Song, G. Kang, L. Pyeon, C. Lim, G.-Y. Lee, T. Park, J. Choi, Systematically optimized bilayered electron transport layer for highly efficient planar perovskite solar cells (η = 21.1%). ACS Energy Lett. **2**(12), 2667-2673 (2017). https://doi.org/10.1021/acsenergylett.7b00888

[S5]Q. Jiang, Z. Chu, P. Wang, X. Yang, H. Liu et al., Planar-structure perovskite solar cells with efficiency beyond 21%. Adv. Mater. **29**(46), 1703852 (2017). https://doi.org/10.1002/adma.201703852

[S6]K. Liu, S. Chen, J. Wu, H. Zhang, M. Qin., Fullerene derivative anchored SnO_2 for high-performance perovskite solar cells. Energy Environ. Sci. **11**(12), 3463-3471 (2018). https://doi.org/10.1039/C8EE02172D

[S7]D. Yang, R. Yang, K. Wang, C. Wu, X. Zhu et al., High efficiency planar-type perovskite solar cells with negligible hysteresis using EDTA-complexed SnO_2. Nat. Commun. **9**(1), 3239 (2018). https://doi.org/10.1038/s41467-018-05760-x

[S8]Y. Zhao, Q. Li, W. Zhou, Y. Hou, Y. Zhao et al., Double-side-passivated perovskite solar cells with ultra-low potential loss. Solar RRL **3**(2), 1800296 (2019). https://doi.org/10.1002/solr.201800296

[S9]Z. Liu, K. Deng, J. Hu, L. Li et al., Coagulated SnO_2 colloids for high-performance planar perovskite solar cells with negligible hysteresis and improved stability. Angew. Chem. Int. Ed. **131**(33), 11621-11628 (2019). https://doi.org/10.1002/ange.201904945

[S10] K. Schutt, P.K. Nayak, A.J. Ramadan, B. Wenger, Y.-H. Lin, H.J. Snaith, Overcoming zinc oxide interface instability with a methylammonium-free perovskite for high-performance solar cells. Adv. Func.Mater. **29**(47), 1900466 (2019). https://doi.org/10.1002/adfm.201900466

[S11] G. Yang, H. Zhang, G. Li, G. Fang, Stabilizer-assisted growth of formamdinium-based perovskites for highly efficient and stable planar solar cells with over 22% efficiency. Nano Energy **63**,103835 (2019). https://doi.org/10.1016/j.nanoen.2019.06.031

[S12] P. Zhu, S. Gu, X. Luo, Y. Gao, S. Li, J. Zhu, H. Tan, Simultaneous contact and grain-boundary passivation in planar perovskite solar cells using SnO_2-KCl composite electron transport layer. Adv. Energy Mater. **10**(3), 1903083 (2020). https://doi.org/10.1002/aenm.201903083

[S13] R. Wang, J. Xue, K.-L. Wang, Z.-K. Wang, Y. Luo et al., Constructive molecular configurations for surface-defect passivation of perovskite photovoltaics. Science **366**(6472), 1509-1513 (2019). https://doi.org/10.1126/science.aay9698

[S14] X. Zheng, Y. Hou, C. Bao, J. Yin, F. Yuan et al., Managing grains and interfaces via ligand anchoring enables 22.3%-efficiency inverted perovskite solar cells. Nature Energy (2020). https://doi.org/10.1038/s41560-019-0538-4

[S15] T. Bu, J. Li, F. Zheng, W. Chen, X. Wen et al., Universal passivation strategy to slot-die printed SnO_2 for hysteresis-free efficient flexible perovskite solar

module. Nat. Commun. **9**(1), 4609 (2018). https://doi.org/10.1038/s41467-018-07099-9

[S16] Q. Jiang, Y. Zhao, X. Zhang, X. Yang, Y. Chen et al., Surface passivation of perovskite film for efficient solar cells. Nat. Photonics **13**(7), 460-466 (2019). https://doi.org/10.1038/s41566-019-0398-2

[S17] P. Cui, D. Wei, J. Ji, H. Huang, E. Jia et al., Planar p–n homojunction perovskite solar cells with efficiency exceeding 21.3%. Nat. Energy **4**(2), 150-159 (2019). https://doi.org/10.1038/s41560-018-0324-8

[S18] P. Wang, R. Li, B. Chen, F. Hou, J. Zhang, Y. Zhao, X. Zhang, Gradient energy alignment engineering for planar perovskite solar cells with efficiency over 23%. Adv. Mater. **32**(6), 1905766 (2020). https://doi.org/10.1002/adma.201905766
